# Cross-country analysis of contextual factors and implementation strategies in under-5 mortality reduction in six low- and middle-income countries 2000–2015

**DOI:** 10.1186/s12887-023-03906-5

**Published:** 2024-02-28

**Authors:** Agnes Binagwaho, Amelia VanderZanden, Patricia J. Garcia, Fauzia Akhter Huda, Mahesh Maskey, Mohamadou Sall, Felix Sayinzoga, Raj Kumar Subedi, Alula M. Teklu, Kateri Donahoe, Miriam Frisch, Jovial Thomas Ntawukuriryayo, Kelechi Udoh, Lisa R. Hirschhorn

**Affiliations:** 1 University of Global Health Equity, Kigali, Rwanda; 2 School of Public Health at Cayetano Heredia University, Lima, Peru; 3 Global Health Department, University of Washington, Seattle, USA; 4 icddr,b, Dhaka, Bangladesh; 5 Nepal Public Health Foundation, Kathmandu, Nepal; 6 The Cheikh Anta Diop University, Dakar, Senegal; 7 Rwanda Biomedical Center, Kigali, Rwanda; 8 MERQ Consultancy PLC, Addis Ababa, Ethiopia; 9 Northwestern University, Chicago, USA

**Keywords:** Under-5 mortality, Implementation research, Evidence-based interventions, Amenable mortality, Low- and middle-income countries, Implementation strategies, Contextual factors

## Abstract

**Background:**

The Exemplars in Under-5 Mortality (U5M) was a multiple cases study of how six low- and middle-income countries (LMICs), Bangladesh, Ethiopia, Nepal, Peru, Rwanda, and Senegal, implemented health system-delivered evidence-based interventions (EBIs) to reduce U5M between 2000 and 2015 more effectively than others in their regions or with similar economic growth. Using implementation research, we conducted a cross-country analysis to compare decision-making pathways for how these countries chose, implemented, and adapted strategies for health system-delivered EBIs that mitigated or leveraged contextual factors to improve implementation outcomes in reducing amenable U5M.

**Methods:**

The cross-country analysis was based on the hybrid mixed methods implementation research framework used to inform the country case studies. The framework included a common pathway of Exploration, Preparation, Implementation, Adaptation, and Sustainment (EPIAS). From the existing case studies, we extracted contextual factors which were barriers, facilitators, or determinants of strategic decisions; strategies to implement EBIs; and implementation outcomes including acceptability and coverage. We identified common factors and strategies shared by countries, and individual approaches used by countries reflecting differences in contextual factors and goals.

**Results:**

We found the six countries implemented many of the same EBIs, often using similar strategies with adaptations to local context and disease burden. Common implementation strategies included use of data by decision-makers to identify problems and prioritize EBIs, determine implementation strategies and their adaptation, and measure outcomes; leveraging existing primary healthcare systems; and community and stakeholder engagement. We also found common facilitators included culture of donor and partner coordination and culture and capacity of data use, while common barriers included geography and culture and beliefs. We found evidence for achieving implementation outcomes in many countries and EBIs including acceptability, coverage, equity, and sustainability.

**Discussion:**

We found all six countries used a common pathway to implementation with a number of strategies common across EBIs and countries which contributed to progress, either despite contextual barriers or by leveraging facilitators. The transferable knowledge from this cross-country study can be used by other countries to more effectively implement EBIs known to reduce amenable U5M and contribute to strengthening health system delivery now and in the future.

**Supplementary Information:**

The online version contains supplementary material available at 10.1186/s12887-023-03906-5.

## Main messages

- These six countries which dropped under-5 mortality faster than their peers between 2000 and 2015 used a number of common implementation strategies to implement evidence-based interventions known to reduce amenable under-5 mortality in low- and middle-income countries.
- These countries used a common pathway to implementation from Exploration to Preparation, Implementation, Adaptation, and Sustainment, with variability reflecting national and subnational contexts and learning during implementation.
- Use of data and stakeholder input to understand and act on contextual factors which can hinder or facilitate implementation, and to choose and adapt implementation strategies to reflect mechanisms of action, can help improve implementation outcomes of these evidence-based interventions.
- Using implementation research to understand how and why countries successfully implement evidence-based interventions is valuable to extract transferable lessons which can inform work to accelerate progress in further reducing under-5 mortality in other countries.

## Introduction

Between 2000 and 2015 and supported by the efforts of the Millennium Development Goals (MDGs), there was an increase in focus and funding towards maternal and child health. This effort contributed to progress in low- and middle-income countries in reducing under-5 mortality (U5M) by 43% globally [1–5]. The commitment to achieving the MDGs spurred countries to introduce and strengthen implementation of evidence-based interventions (EBIs) known to reduce U5M directly through prevention or treatment of leading causes of death, through health interventions which reduced U5M risk, and through broader public health system strengthening that supported this work to reduce U5M [6]. Despite these well-known health system-delivered EBIs being used to reduce U5M, success in implementing these interventions was not uniform [7]. Much of the published work to understand these efforts has focused on overall coverage and effectiveness, and less on how and why countries succeeded in the implementation strategies used and contextual factors which helped or hindered this work [8, 9].

Implementation research is “the scientific study of the use of strategies to adopt and integrate evidence-based health interventions into clinical and community settings to improve individual outcomes and benefit population health” [10]. This method of study offers tools that can help understand and create the transferable knowledge needed to support learning between countries which have been more and less successful in EBI implementation [8, 11, 12]. The Exemplars in U5M project used implementation research methods to conduct mixed methods case studies to understand how and why six countries, Rwanda, Nepal, Senegal, Bangladesh, Ethiopia, and Peru, were able to lower U5M more than other countries from similar regions and with comparable economic growth [13, 14]. From 2000 to 2015, these countries dropped U5M by more than 50%, and all but Senegal achieved MDG4, defined as more than two-thirds decline in U5M during that period [5]. The case studies focused on the implementation of the health system-delivered EBIs known to reduce amenable U5M, defined as deaths preventable through quality in healthcare delivery [15]. In every country we studied, work in other sectors – including female empowerment and education, economic growth, and water, sanitation, and hygiene – also served as facilitators or barriers to the implementation strategies or their adaptation, in and beyond the health system, and the overall impact of the EBIs in reducing U5M. While we recognize their importance, understanding the exact contribution was not part of our study. Further, while we also explored other interventions outside the health system EBIs known to reduce the risk of major causes of U5M as well as improve survival, in-depth analysis of how these were implemented was beyond the scope of this project.

For this paper, we conducted a cross-country analysis from the country case studies to understand similarities and differences across the six countries. Using the lens of a framework for implementation we adapted to in order to look at the decision pathways from exploration to preparation, implementation, adaptation, and sustainment (EPIAS), we strove to understand how the countries mitigated barrier or leveraged facilitating contextual factors through the choice, implementation, and adaptation of implementation strategies, as well as the implementation outcomes of the work to implement health system-delivered EBIs to reduce amenable U5M. As we reach the midpoint of the Sustainable Development Goals period, many countries are continuing to work to achieve gains in reducing under-5 and neonatal mortality. We hope the results of this study will be of value to implementers and policymakers looking to accelerate work to strengthen implementation of existing EBIs for future interventions to reduce amenable child mortality in different contexts and to contribute to strengthening public health system delivery now and in the future.

## Methods

### Case study design

**Fig. 1:**
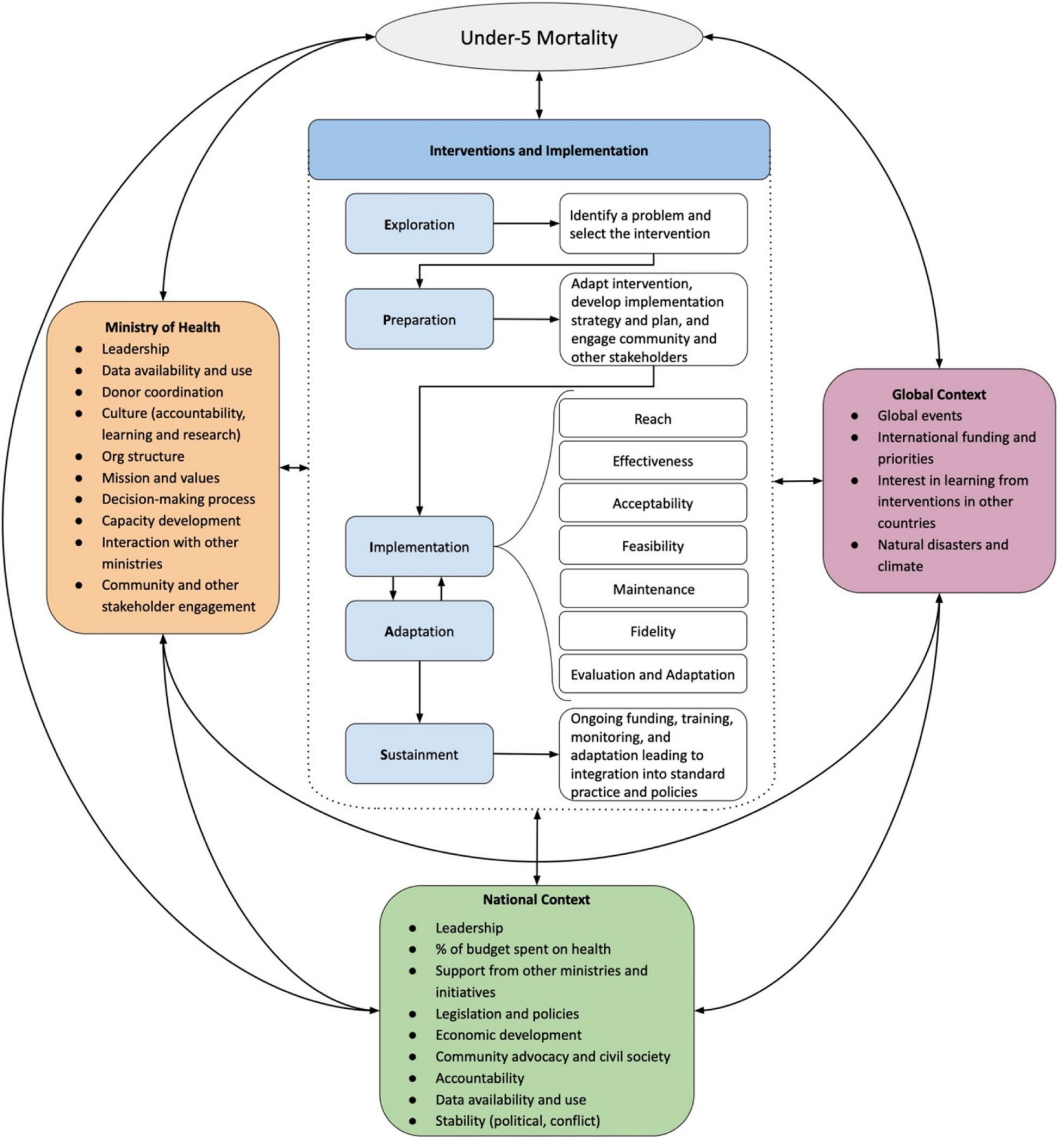
Hybrid framework for understanding health system-delivered evidence-based interventions to reduce under-5 mortality in low- and middle-income countries

The details of the Exemplars in U5M project are described on the Exemplars website (https://www.exemplars.health/topics/under-five-mortality), with methodological details of the country case studies published elsewhere [15]. Briefly, six low- and middle-income countries – selected to represent a range of locations and population sizes – were identified as having experienced greater U5M drops between 2000 and 2015 in relation to the gross domestic product per capita than countries in their regions with similar socioeconomic development. The mixed methods case studies were informed by a hybrid implementation research framework designed for the Exemplar study [15]. This framework built on existing implementation research frameworks and EPIAS (Exploration, Preparation, Implementation, Adaptation, and Sustainment) steps to understand the implementation pathways, contextual factors at the global, national, health system, and community level, and implementation outcomes including appropriateness, feasibility, acceptability, fidelity, effectiveness, equity, and sustainability (Fig. 1) [15].

#### Evidence-based interventions

We reviewed existing literature and guidelines to identify EBIs known to reduce the most common causes of amenable mortality among neonates and children under 5 in low- and middle-income countries during the study period [16, 17] (Additional file 1).

#### Data collection

Between 2017 and 2020 we analyzed publicly available quantitative data on mortality, causes of death, and EBI coverage over the study period. We also conducted desk reviews of reports, policy documents, and published articles and other gray literature describing strategies, contextual factors, implementation outcomes, and policies related to these health system-delivered EBIs. Finally, we conducted 104 key informant interviews across the six countries (range 11 in Ethiopia to 23 in Senegal) with current and former ministry of health officials, implementers, donors, and other global, national, or subnational actors using a semi-structured interview guide based on the implementation research framework. The guides were adapted to each country and designed to understand the EBI implementation process, from exploration and preparation, through implementation, adaptation, and sustainment (EPIAS) (see Additional file 2 for a key informant interview guide). The key informants (KIs) were chosen purposively to represent a variety of viewpoints and experiences during the study period of 2000–2015. The selection of KIs was not intended for saturation but to cover a range of EBIs and was limited by time and resources. Resource limitations precluded inclusion of community and frontline providers, although a number of key informants served as a provider of these EBIs prior to their current position. The interviews were conducted by phone or face-to-face by the U5M project principal investigators (AB and LRH), lead country partners (PJG, FAH, MM, MS, FS, RKS, AMT), and project staff (KU). Each interview’s duration was approximately 1 h. The country cases were designed and conducted using the same methodology and core tools for data collection and analysis to facilitate a cross-case analysis.

To understand the success or challenges of the EBI implementation, we focused on implementation outcomes including acceptability, appropriateness, effectiveness, feasibility, fidelity, coverage (reach), sustainability, and equity where available (Table 1) [18]. While not typically an implementation outcome, we included coverage as it is critical to achieving drops in U5M. In feedback sessions we had for each country as part of the case study development process, we sought input from country partners and health system stakeholders for consensus on the identification of outcomes. There were challenges with data gaps and limitations for implementation outcomes both through primary and secondary sources. To support the qualitative acceptability data, we also used coverage and reach data. We recognize however that other factors impact acceptability, for example, including access such as through geography.

**Table 1:** Key terms and definitions

Term	Definition
**Implementation strategies**	The approaches and methods used to adopt, implement, and sustain EBIs [19].
**Contextual factors**	The global, national, MOH, and community/family/individual level factors which influence the success and failure of the implementation and effectiveness of the EBI [20]. These can also influence U5M rates more broadly. They can be addressed if a barrier, leveraged if a facilitator, inform implementation strategy choice or adaptation, or be acknowledged representing a factor not able to be addressed or adapted to through a strategy.
**Implementation outcomes**^a^	The results of the implementation of the EBIs which represent “how well” implementation strategies were executed as well as the interaction between the strategies and contextual factors.
**Appropriateness**	The perceived fit of the intervention (as originally conceptualized and as implemented) to address a particular cause of death or for a specific setting.
**Acceptability**	The perception of stakeholders that an EBI is agreeable and is typically based on qualitative or user experience and attitudes^b^ and could include satisfaction with the type of services [19].
**Coverage**	The geographic or population spread of an EBI.
**Cost**	The incremental cost of the implementation of the intervention.
**Effectiveness**	The evidence of change in the targeted cause of disease and death.
**Equity**	The coverage or reach of EBIs among different subpopulations defined by factors such as wealth, gender, and geography.
**Feasibility**	The practicability of an EBI and the extent to which an intervention (as originally conceptualized and as implemented or as adapted) can be or has been successfully implemented, used, or carried out within a given setting.
**Fidelity**	*Fidelity to the implementation*: the degree to which an intervention was implemented as it was prescribed in the original plan or as it was intended by the program developers (before implementation and after adaptation). *Fidelity to the EBI:* the degree to which the intervention was delivered as defined by guidelines and standards of care.
**Reach**	The extent to which an EBI reached everyone intended (see also equity).
**Sustainability**	The extent to which a newly implemented treatment is maintained or institutionalized within the ongoing and stable operations of a service setting. This measures if evidence of sustainability was seen, such as coverage rates maintained, or strategies implemented to support sustainment.

*EBI* evidence-based intervention, *MOH* ministry of health, *U5M* under-5 mortality

*Note*: Key terms are based on the literature sources and adapted as needed

^a^Outcomes defined here are adapted from Proctor et al. 2011 [18]

^b^To support the qualitative acceptability data, we also used coverage and reach data, while recognizing that other factors impacted acceptability including geographic access

#### Quantitative analysis

We used equity plots produced by the International Center for Equity in Health and data on equity gaps collected and analyzed from Demographic and Health Survey data for each country, for coverage of selected EBIs including vaccinations and facility-based delivery (FBD), and to explore changes in countries’ equity gaps over time across region, wealth quintile, and sex.

#### Qualitative analysis

For the country-specific analysis, we used a sequential explanatory mixed methods approach; we used coverage data to inform qualitative questions [21], and used directed content analysis of the KI interviews based on the hybrid framework [22, 23]. Following the close of the interview, notes were combined and audio recordings (if permitted) were used to clarify areas as needed. We analyzed the key informant interviews to expand on the literature review, identifying the contextual factors and implementation strategies to understand how and why countries reached their quantitative results. Using the framework, we created an initial set of codes for EBIs, contextual factors, and implementation outcomes. We coded manually and added new codes as we identified new concepts, contextual factors, or implementation strategies. We used inductive and deductive approaches for thematic coding of interviews; this was reviewed by one of the principal investigators for accuracy, with discussion for differences. Guided by the framework we also extracted evidence from the quantitative and qualitative data sources to explore other implementation outcomes such as acceptability, adoption, and equity. We analyzed and synthesized the findings and presented them for review during a convening of in-country stakeholders for feedback and validation.

### Cross-case analysis and synthesis

We performed the cross-case analysis using multiple case studies methodology, in which common research questions unite the cases, facilitating a greater understanding of the set of cases from both commonality and uniqueness [24–26]. We reviewed each case study and extracted the contextual factors, implementation strategies, and implementation outcomes by EBI for each country into an excel database, creating matrices to identify those which were more or less common. We used the case studies to identify cross-cutting contextual factors that were facilitators or barriers, as well as those that could be either a barrier or facilitator depending on local context, or those not identified, as contextual factors to EBI implementation. We developed a matrix of all implementation strategies identified across the countries and EBIs and used it to assess whether selected strategies were implemented successfully, implemented with some success, or not implemented at all. We defined “cross-cutting” implementation strategies as those implemented in at least three of the six countries. We reviewed implementation outcomes identified in the case studies to explore where EBI implementation was more, or less, successful across the six countries.

### Ethical considerations

The study was determined to be non-human subjects research by the Rwandan National Ethics Committee and Northwestern University reflecting the scope and focus, and each of the country case studies were reviewed and approved by in-country Ethics Review Committees (Bangladesh, PR-18074**;** Ethiopia, PM23/281; Nepal, 165–2018; Peru, 104,276; Rwanda, 132/RNEC/2017; Senegal, SEN18/33). Interviewees were informed about the goals and structure of the project, and verbal informed consent for participation was obtained from all interview participants.

## Results

### Common pathway for implementation of interventions

We found that the six countries implemented many of the same EBIs, often using similar strategies with adaptations to local context and disease burden and a common pathway of EPIAS stages. They often went through similar decision-making processes, identifying and leveraging facilitating contextual factors or mitigating identified barriers. While the process used many of the same strategies across EBIs and countries, the decision-making and contextual differences also contributed to adaptation of how these were implemented, as well as some strategies which were more specific to national or subnational contexts. For instance, difficult geographic access was a challenge for EBI implementation including delivery in healthcare facilities for women who lived in mountainous areas in countries such as Nepal and Bangladesh. Both countries responded to this barrier by integrating a cadre of skilled births attendants into the existing community health worker (CHW) system who could reach provide delivery services to the women, and Nepal added another strategy of cash transfers for transport for pregnant women who delivered in health facilities. In Ethiopia, the community-based delivery of EBIs was also implemented, with use of health extension workers who reached and built trust with underserved pastoralist communities with limited access to healthcare.

During the Exploration phase, all countries used data to understand the burden of disease, determine if the EBI was appropriate for implementation at that time, and to establish whether EBI adaptation was needed. The type of data used (such as previous analysis from the country or global evidence, newly analyzed from existing routine data, or newly collected data) differed by EBI and by country. For example, in Bangladesh, icddr,b, an academic research institution, supported the government with pilot testing and data collection. This existing research capacity allowed for ongoing data analysis to inform the needs of interventions and their adaptation. It also encouraged policymakers to rely on national data collection and analyses. Further, this allowed Bangladesh to innovate based on in-depth understanding of relevant topics and local context. In Nepal, the Ministry of Health (MOH), in close collaboration with implementing partners, adopted EBIs after conducting small-scale testing to confirm appropriateness and acceptability. The Nepal Health Research Council assessed quality of the findings from research conducted in a small geographic area and, if found a good fit, recommended the integration of tested interventions into national policy and planning. Rwanda, on the other hand, more often leveraged existing data, routinely collected from patients in healthcare facilities. According to one key informant, Rwanda’s investment in information technology and systems, improving data availability at the local level, meant that *“All the data went through that platform, and each level can see that data and can analyze themselves (over time). This platform can help them to see how (things are working).”*

During the Preparation stage, countries employed a number of common strategies. These strategies were used to inform understanding and to choose implementation strategies when possible to directly address contextual barriers or leverage facilitating factors to effectively implement the chosen EBIs. Common strategies included use of data, partnering with donors and collaborators (both within the health sector and across ministries and implementing partners), and engaging with stakeholders from national to community levels. For instance, Rwanda employed a public/nongovernmental organization/faith-based organization partnership strategy, signing formal agreements to provide the same package of health services and reports as the one provided by the public sector. In return, these health facilities received the same supervision and government support for salaries, equipment, and infrastructure as the public health facilities. This collaboration increased the number of district hospitals and health centers available to the population. All the countries also effectively used data to understand disease burden to prioritize work and resources.

Common strategies used by countries during the Implementation stage included engagement and education of community; integration of EBI policies and protocols into standards of care; activation of monitoring and evaluation systems; and training of personnel and stakeholders. In Peru, for example, the MOH developed and implemented a national sexual and reproductive health strategy beginning in 2004, promoting FBD as one of the country’s main strategies to reduce maternal mortality. Training and other human resources strengthening activities such as supportive supervision facilitated implementation of new EBIs or expansion of coverage for existing ones. In the case of FBD, this included national-level training for providers. In Senegal, community engagement and use of data systems were important in implementing strategies to reduce malaria. According to KIs, a significant strategy associated with success in Senegal’s malaria program was community engagement through awareness-raising campaigns that involved a variety of door-to-door and community-wide outreach activities. Routine data collection and surveys, including the Demographic and Health Survey and the Malaria Indicator Survey, were used to monitor important indicators such as care-seeking rates and preferences for available malaria treatment options. All countries used the existing community health system to implement EBIs, although the emphasis ranged between countries with the highest in Rwanda, Nepal, Ethiopia, and Bangladesh.

Countries continued to use data to direct work on implementation outcomes including reach and fidelity and ongoing stakeholder engagement to identify where Adaptations in existing strategies or where new strategies were needed at a national or subnational level. For example, in Ethiopia, coverage data helped in recognizing gaps in newborn care. Ethiopia used this information to adapt the services provided by its health extension workers, putting a priority on neonatal mortality by adding in community-based newborn care to the curriculum.

Less uniform between countries and across EBIs was use of strategies in laying the groundwork to ensure Sustainment – that effective implementation strategies and EBI delivery would be maintained. In addition, KIs reported that donor funding was a potential threat to the sustainment of U5M initiatives, due to countries being overly dependent on donor funding. For example, Senegal chose strategies including leveraging donor support and increasing government funding to try to influence sustainment, with total health expenditure per capita increased from $22 in 2000 to $36 in 2015. In Nepal, there was an ongoing reliance on donor funding despite efforts to increase government financing and leverage donor support. This made sustainment a challenge as Nepal’s economic status increased and donor funding priorities reoriented to other countries.

### Contextual factors

Facilitating factors for EBI implementation found across many or all of the countries included culture of data use, stakeholder engagement, an existing CHW structure, national priority for health and primary care, leadership and governance and a culture of accountability, donor funding availability and aligned priorities with national agenda, and economic development (Table 2). Contextual factors that were barriers (or could be, depending on local context) included geography, health system structure and strength, and culture and beliefs around EBIs. Countries often chose or adapted strategies to leverage facilitators or directly or indirectly address barriers. Below we discuss differences in examples of important contextual factors, both facilitators and barriers. More examples are in Additional file 3 as well as the full case studies available at https://www.exemplars.health/topics/under-five-mortality [27–31].

**Table 2:** Key contextual factors across the six countries which were facilitators, barriers, or both

Contextual factor	Bangladesh	Ethiopia	Nepal	Peru	Rwanda	Senegal
Donor funding priorities and availability	**+**	**+**	**+**	**+**	**+**	**+**
Global implementation tools (such as guidelines)	**+**	**+**	**+**	**+**	**+**	**+**
Conflict	**–**	**–**	**N/I**	**N/I**	**N/I**	**–**
Culture of donor and partner coordination	**+**	**+**	**+**	**+**	**+**	**+/−**
Economic growth	**+**	**+**	**+**	**+**	**+**	**+**
Financial commitment to the health sector	**+**	**+/−**	**+/−**	**+**	**+/−**	**+/−**
Geography	**+/−**	**–**	**–**	**N/I**	**+**	**–**
Health insurance	**–**	**+**	**–**	**+**	**+**	**+/−**
Health systems structure and strength	**+**	**+/−**	**+/−**	**+**	**+**	**+/−**
In-country research capacity	**+**	**N/I**	**+**	**+**	**N/I**	**+**
Leadership and governance and a culture of accountability	**+**	**+**	**+**	**+/−**	**+**	**+**
National priority for health and primary care	**+**	**+**	**+**	**+**	**+**	**+**
Non-health national infrastructure and systems strengthening	**+**	**N/I**	**+**	**+**	**+**	**N/I**
Preexisting culture and capacity of data use	**+**	**+**	**+**	**+**	**+**	**+**
Strong preexisting community health system and structure including CHWs	**+**	**+**	**+**	**+**	**+**	**+**
WASH	**+**	**+**	**+**	**+**	**+**	**+**
Culture and beliefs	**+/−**	**–**	**–**	**–**	**+**	**+**
Female empowerment and education	**+**	**+/−**	**+**	**+**	**+**	**+**
Reproductive rights	**+**	**+**	**+**	**N/I**	**+**	**+**
Stunting	**+**	**+**	**+**	**+**	**–**	**+/−**

**+** Facilitating contextual factor **-** Hindering contextual factor **+/−** Both a facilitating and hindering contextual factor **N/I** Not identified as having an impact

*CHW* community health worker, *KI* key informant, *MOH* ministry of health, *U5M* under-5 mortality, *WASH* water, sanitation, and hygiene

#### Preexisting culture and capacity of data use

We found the pre-existing culture and capacity of data use for evidenced-based decisions was a facilitator in all countries. In Senegal, this contextual factor was important for making data use an effective strategy and facilitating the design, planning, and piloting of EBIs tailored to the local context before they were scaled up. According to one KI, the pre-existing culture of data use enabled decision-makers to use pilot data to “s*ee the costs of implementation; see where to scale-up; evaluate all the needs that must be available first, quantify everything; [and] know which particular actors must be trained and supervised.”* Bangladesh leveraged its culture and capacity of in-country data generation and data use for understanding gaps, implementing, and adapting strategies. This was facilitated through researchers from in-country research institutions such as icddr,b and others participating in regular meetings with decision-makers.

#### Strong preexisting community health system and structure including CHWs

A strong community health system and structure including CHWs was an important facilitator across many of the countries. For example, Ethiopia’s comprehensive community health system, the Health Extension Program, was identified by several KIs as a key facilitator in the country’s efforts to implement EBIs to reduce U5M. An Ethiopian KI explained that *“it is difficult to say there is one and only one initiative but the major umbrella I would say could be the Health Extension Program into which [was] imbedded management of childhood illness, and the newborn community-based initiatives along with integration of child immunization program into the [Health Extension Program] and this … encompasses both rural and urban communities. That is one area that has contributed much of the reduction to under-5 mortality.”* The Health Extension Program increased access to health education and promotion, prevention, and delivery services, particularly in rural areas – and became a platform to introduce new services such as integrated community case management. Similar findings included the leveraging of the CHWs in Rwanda through the Binome program and in Nepal through the Lady Health Workers or female community health volunteers. A KI in Nepal said that the female community health volunteers were important in contributing to major achievements in the health sector, stating that they *“were behind every major changes and achievements in the health sector and I consider them to be one of the main reasons behind the U5 mortality drop.”*

#### Leadership and governance and a culture of accountability and, closely related, national priority for health and primary care

We found leadership and governance and national priority for health including a focus on primary care and linking to accountability to be facilitators in each of the six countries. For example, in Rwanda and Nepal, the commitment of leadership to access to healthcare as a human right of the people is enshrined in the constitution. A high national priority of “reaching the unreached” was a core theme repeated by several KIs. In Nepal this commitment to healthcare allowed the work to reduce U5M to continue during the country’s civil war because all sides protected the health sector programs. In Peru, the Roundtable for the Fight Against Poverty, a multisectoral initiative that set a national anti-poverty agenda, facilitated national leadership and prioritization and increased the culture of accountability for implementation of EBIs. One KI explained, *“the Roundtable is reviewing budget and implementation issues and looking at the problems that are in regions, and I think it also helps the same actors who execute to be able to feel observed and seen, and not just do what they want without accountability and transparency.”*

Factors could be either a barrier or facilitator, depending on the country. A factor could be strong and leveraged in one place while weak and need to be addressed in another, while not really affecting implementation elsewhere. We identified a number of contextual factors shared across many of the countries which were barriers, and which countries worked to directly address or accommodate through adaptation of strategies. For example, we found *culture and beliefs* around healthcare was a barrier factor that needed to be overcome in some places (Table 2). In Peru, cultural practices in many areas meant that pregnant women who lived in rural Andean regions preferred to deliver in vertical position. To increase FBD, cultural sensitivity to this practice was integrated into the delivery protocols (*strategy: adaptation during implementation)*. In Ethiopia, the pastoralist communities believed that delivery was a normal process which did not necessarily require delivery in health facilities. In addition, it was not culturally acceptable for postpartum women to leave homes within hours after delivery, creating a barrier to accessing postnatal care in health facilities. To reduce the risk of life-threatening complications during perinatal period, health extension workers were trained to provide postnatal care at home (*strategy: building on CHW program and community-based care delivery)*. In Senegal, some pregnant women still relied on traditional norms, such as hiding early pregnancies, delaying uptake of healthcare services including antenatal care visits. In response, the country leveraged its preexisting community health system and structure including CHWs, introducing a new cadre of CHWs (*strategy: building on CHW program and community-based care delivery)*, locally known as *bajenou gokh,* who were culturally respected older women. The *bajenou gokh* saw to health promotion including early detection of pregnancy and encouragement of care-seeking behavior, such as antenatal care visits and FBD.

#### Geography

In many countries, geographic barriers were addressed through strategies to facilitate access to healthcare services for people living in hard-to-reach areas. For instance, in Senegal, a cadre of home-based care providers, known as *dispensateurs de santé à domicile*, was introduced to provide basic healthcare services such as testing and treatment of malaria for underserved people living in hard-to-reach areas. In Ethiopia, although there was scale up of the health extension program to deliver EBIs to underserved people across the country, coverage was still low in some regions particularly where most pastoralist communities lived. In Nepal, people who lived in hard-to-reach areas had limited access to health services. The country prioritized conducting small-scale testing of EBIs such as measles vaccination and malaria interventions primarily in these areas and, after having effective findings from the testing, the interventions were scaled up to other regions.

Many implementation strategies were mapped to specific contextual factors across different EBIs. For example, the strategies chosen to focus on equity reflected contextual factors underlying inequity such as existing gaps in economic development (poorer versus wealthier populations) and geography (including both urban/rural and hard-to-reach areas). The specific strategies which were then applied differed between countries and included subsidizing or providing free care, beginning implementation in poorer areas, or targeting focused interventions in hard-to-reach areas, depending on the contextual factors associated with existing inequity.

### Common implementation strategies

The six countries often chose or adapted implementation strategies to address or leverage identified contextual factors before or during implementation. Important strategies implemented across all or most of these countries included data use for decision-making, multisectoral collaboration, building and leveraging existing primary healthcare systems to integrate new EBI delivery into existing systems including CHWs, national leadership and accountability for EBI implementation, integrating the EBI into national protocols and policies, and community engagement and education (Table 3). Countries also selected strategies to address some contextual factors which were barriers, directly through the health sector or by leveraging national level work led by the ministry of health and other sectors such as infrastructure development, female empowerment, nutritional interventions to reduce stunting, water, sanitation, and hygiene, and broader work in strengthening human resources for health. This approach was important in adapting strategies addressing barriers, for example through access-focused strategies to address cultural barriers and improve acceptability. Below we discuss examples of key cross-cutting implementation strategies as well as some that were implemented with more variable success. More examples are in the full case studies available at https://www.exemplars.health/topics/under-five-mortality [27–31].

**Table 3:**
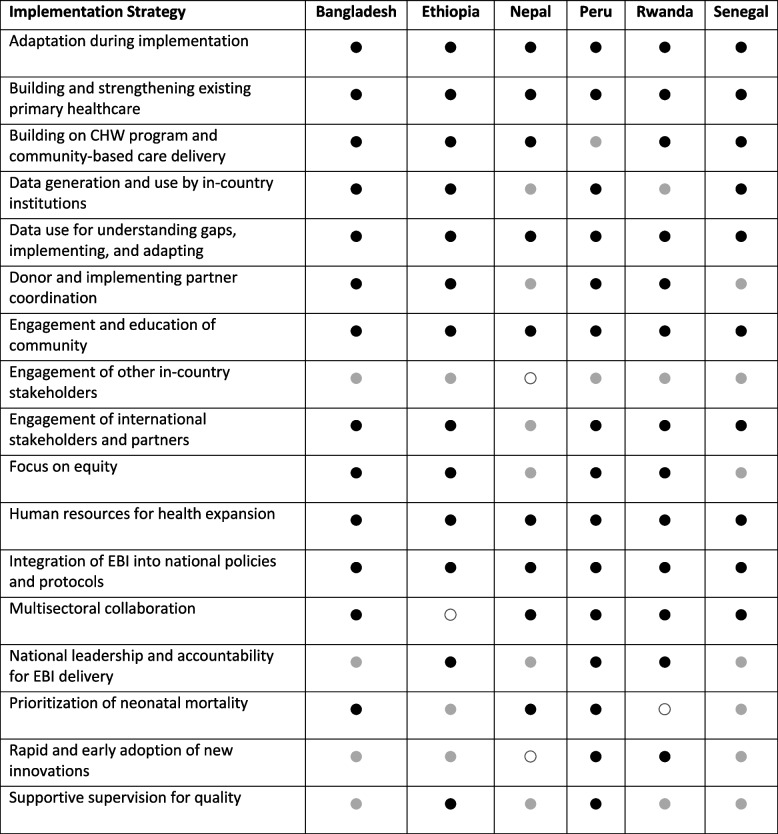
Selected common implementation strategies in the reduction of amenable under-5 mortality through health system-delivered evidence-based interventions

● Strategy effectively implemented

Strategy implemented with variable success

○ Strategy not implemented

*CHW* community health worker, *EBI* evidence-based intervention, *KI* key informant, *U5M* under-5 mortality

Countries leveraged implementation strategies such as building and strengthening existing primary healthcare based on the contextual factor of strong preexisting community health system and structure including CHWs. The strategies of rapid versus phased scale-up reflected factors including donor funding priorities and availability, national priority for health and primary care, and geography. Finally, countries leveraged partner support (for example for evidence generation, cold and supply chain strengthening, and implementation), reflecting gaps in health systems structure and strength, culture and beliefs, and preexisting culture and capacity of data use.

#### Multisectoral collaboration

Multisectoral collaboration, including across ministries and particularly for interventions to address barriers, was consistently important for implementation success. This strategy was also combined in some of the countries with strong coordination of donors who often funded much of the initial EBI implementation to implement following national priorities and guidelines. In Senegal, partners across sectors and ministries collaborated to set the policy agenda and priorities for national maternal and child health programming, with the Ministry of Economy, Finance, and Planning a key partner to avoid duplicating efforts and increase efficiency in planning for implementation. A KI explained that collaborative planning was essential, *“because there are many stakeholders, we need to pool resources according to needs and priorities. If two stakeholders are in the same area, we are better pooling them instead of coming in the same area do the same work without knowing each other’s programs.”* Rwanda used multisectoral collaboration as an implementation strategy through the Social Cluster (made up of Ministries of Health, Education, Local Government, Agriculture, and Gender), which worked to address overlapping issues, such as malnutrition, teenage pregnancy, and gender-based violence. Peru used multisectoral collaboration for sustained efforts, such as the Roundtable for the Fight Against Poverty, which specifically targeted maternal and child health indicators and remained active throughout government transitions, with collaboration between civil society, the public sector, and nongovernmental organizations.

#### Building on CHW program and community-based care delivery

The adaptation or expansion of the existing community-based care delivery systems (a facilitating factor) and building on CHW programs was an important strategy in five countries, though less so in Peru. Community health workers have been utilized in Peru for decades but tended to have little training, working as volunteer health promoters, and were used with variable effectiveness for a few EBIs. Ethiopia leveraged its Health Extension Program, establishing health extension workers to implement multiple key community-level EBIs through demand generation (via community engagement and education) as well as care delivery (including vaccines, antenatal care, and FBD). Senegal leveraged its strong community-based care delivery system with multiple cadres of CHWs to engage and educate communities and provide direct care delivery. This strategy helped to increase acceptability as well as facilitate broader scale-up of interventions.

#### Focus on equity

Prioritization of equity was an important strategy across countries, but took different forms. In Bangladesh, this strategy was used in integrated management of childhood illness, with areas with higher U5M targeted at the start of the phased roll-out. In Peru, a focus on equity meant the country prioritized introducing new vaccines, including rotavirus and pneumococcal, into the poorest areas first before national rollout. However, even where countries experienced overall reductions in U5M, there was variability subnationally in every country, with some areas or groups lagging behind in the reduction in U5M and in coverage of some EBIs [32]. Subnational variability in amenable U5M reductions was observed for disease incidence, EBI reach – often related to contextual factors such as culture, geography (by region and urban/rural) – and socioeconomic status. Across the six countries, U5M and neonatal mortality were higher in rural areas and among the poorest [33–44].

Even when the EBI implementation strategies were carefully planned, they were not always effective. We found that countries had successes when they recognized and diagnosed the challenges and addressed them through strategy adapted to context (for example, Peru’s modification of maternal waiting homes, discussed further below) or directly addressing the barrier (such as home-based care delivery when geography limited access). Implementation strategies chosen by countries to address quality – such as supportive supervision – were implemented with more variable success. Supportive supervision was not always done routinely (Senegal), or sufficiently (Rwanda and Ethiopia), or with high quality (Nepal). In Senegal, it was noted that supervision was variable due to its reliance on donor resources. In Rwanda, lack of supervision within the CHW program was one of the reasons attributed to challenges to identifying cases of malnutrition in the community using existing CHW systems. In Ethiopia, health extension workers received quarterly supportive supervision visits. However, KIs identified ensuring continued supportive supervision for specific interventions such as integrated community case management through integration into the routine supervision as a challenge to this strategy’s effective implementation.

#### Evidence-based intervention type and implementation strategy

The use of a given implementation strategy reflected the nature or complexity of systems and skills needed to deliver the EBI. For example, some implementation strategies supported EBIs delivered at facilities (e.g. facility staff training in management and monitoring and evaluation), while other strategies could be implemented for community-delivered EBIs (e.g. the strategy of community-based care delivery); still other strategies were agnostic of intervention delivery (e.g. multisectoral collaboration and focus on equity). Preventative health system EBIs such as vitamin A supplementation, insecticide-treated bed net distribution, and intermittent preventive therapy for groups at high risk for malaria exposure could be delivered both in the community and in facilities. For these types of EBIs, dual strategies were often chosen to take advantage. These differentiations can be understood by different types of EBIs, in the following categories: vaccination, preventative (neonatal or children under 5 broadly), and curative (again, neonatal or children under 5 broadly) (Table 4).

**Table 4:** Evidence-based intervention type and relevant implementation strategies

Intervention type	Intervention characteristics	Relevant implementation strategies
Vaccination, e.g., pneumococcal conjugate, rotavirus, measles, pentavalent, tetanus toxoid	Could be delivered within the community and in facilities. Required mass administration of vaccines.	• Community-based care delivery • Leveraging CHWs and other existing systems • Rapid scale-up • Cold chain and supply chain strengthening
Other neonatal preventative interventions, e.g., antenatal care, prevention of mother-to-child transmission of HIV, facility-based delivery and associated practices, post-natal care	Mainly delivered at facilities.	• Human resources for health expansion • Engagement and education of community • Prioritization of neonatal mortality
Other preventative interventions targeting children under 5, e.g., vitamin A supplementation, insecticide-treated bed nets, indoor residual spraying, intermittent preventative therapy for high-risk groups	Could be delivered within community and at facility. Required mass administration of drug or distribution.	• Community-based delivery • Rapid scale-up • Engagement of international stakeholders and partners • Engagement of in-country stakeholders
Curative neonatal interventions, e.g., BEmONC and CEmONC, neonatal resuscitation, neonatal intensive care units, neonatal sepsis management	Complex, facility-based, required higher-level skills, training, and supplies.	• Training • Supportive supervision for quality • Integration of EBI into national policies and protocols • Prioritization of neonatal mortality
Curative interventions targeting children under 5, e.g., facility- and community-based IMCI, anti-retroviral therapy for children	Could be delivered within community and at facility.	• Community-based care delivery • Engagement and education of community

*BEmONC* Basic emergency obstetric and newborn care, *CEmONC* Comprehensive emergency obstetric and newborn care, *CHW* community health worker, *EBI* evidence-based intervention, *IMCI* integrated management of childhood illness

### Implementation outcomes

We were able to find some qualitative and less often quantitative evidence for some implementation outcomes including acceptability, appropriateness, coverage (reach), feasibility, and equity for some of the EBIs and countries. We identified fewer results for other outcomes including cost, fidelity (including quality), and sustainability. Below we discuss findings for outcomes of acceptability, coverage (reach), equity, and sustainability. See Table 5 for more detail on additional outcomes.

**Table 5:** Selected implementation outcomes and examples

Implementation outcome	Examples
Appropriateness	We found appropriateness was high across the six countries in EBIs chosen including vaccination, FBD, and facility- and community-based IMCI. For facility-based and community-based IMCI, the decision to implement reflected identified need through disease burden, gaps in coverage of the relevant preventive and curative interventions, and the need for an integrated approach shown by expert opinion. Other EBI selections reflected disease burden when they were first introduced, such as PCV and PMTCT.
Feasibility	We found feasibility was high where countries had leveraged and integrated EBIs into existing systems (ex. primary care, supply chain) while also strengthening those systems. It was more variable for EBIs that were not integrated into existing systems. For example, feasibility for community-based IMCI was high in Bangladesh. The country leveraged support from partners including WHO and UNICEF for trainings and training guidelines, and conducted phased scale-up with small-scale testing before national roll-out. Conversely, despite efforts to strengthen systems when integrating a new EBI of neonatal intensive-care units (NICUs), in Ethiopia by 2015 only 49% of NICUs were functional.
Fidelity	Evidence of fidelity, defined as the delivery of the EBI as planned and according to national standards, was not found for many of the interventions. Where there were data, it was generally at a local level. For facility-based IMCI, data from Rwanda, Nepal, Ethiopia showed low fidelity with just 2% (Nepal, 2015; Rwanda, 2007) to 5% (Ethiopia, 2014) of children assessed by healthcare providers for general danger signs per the IMCI protocol during consultations. This low fidelity was associated with challenges in strategies such as supportive supervision and training.
	For pentavalent vaccination, we found data only for Bangladesh and Senegal, where fidelity was low, and associated with challenges to strategies such as supervision and health systems strengthening. In Senegal low fidelity due to in part to faults in the cold-chain with vaccines exposed to temperatures outside 2–8 degrees Celsius.

*EBIs* evidence-based interventions, *FBD* facility-based delivery, *IMCI* integrated management of childhood illness, *KI* key informant, *PCV* pneumococcal conjugate vaccine, *PMTCT* prevention of mother-to-child transmission of HIV, *U5* under 5

Countries’ choice of some implementation strategies varied depending on what outcomes they prioritized, as well as the existing contextual factors that needed to be overcome or could be leveraged. For example, geography, cultural barriers to acceptability, and human resource capacity were identified barriers to implementation in many of the countries. Countries often used the strategy of community-based delivery through CHWs or campaigns when populations were more dispersed geographically to achieve reach. Additionally, to achieve effectiveness, this work needed to be accompanied by strategies to strengthen quality through supportive supervision and focus on equity, and interventions to address weak health systems. The strategy of leveraging CHWs as trusted agents to communities was also important for overcoming the barrier of culture and beliefs and achieving the outcome of increased acceptability in countries including Senegal, Ethiopia, and Bangladesh.

#### Acceptability

Acceptability was generally measured by uptake, with variability depending on the EBI and subnational context. Strong community and stakeholder engagement was often associated with higher reports of acceptability. For example, in Nepal, behavior change communications, school-based outreach, and celebrity engagement were all strategies used to increase acceptability of insecticide-treated bed nets. Countries also leveraged acceptability of similar EBIs such as with the rotavirus vaccine in Senegal. According to a KI, “*The advantage is that people have confidence in immunization programs and even when there is a new introduction...they accept to take it.*” Adaptation of strategies was also done to increase acceptability. In Nepal, acceptability of and demand for FBD increased when pregnant women were given conditional cash transfers and transportation vouchers to travel to health facilities. Peru reviewed data on FBD coverage and recognized the need to adapt how FBD and maternal waiting homes were implemented to increase acceptability. They changed facility infrastructure, allowed family members and traditional healers to accompany women, and allowed traditional birthing techniques such as vertical delivery as well as existing techniques.

#### Coverage (reach)

**Fig. 2:**
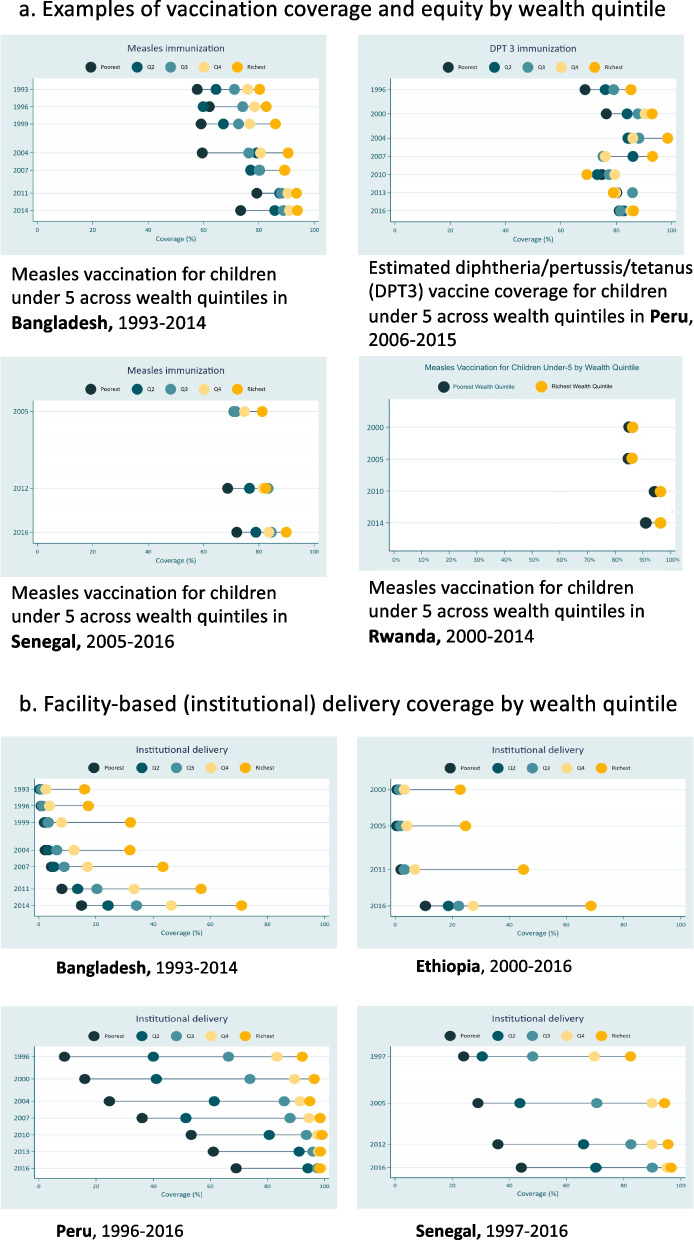
Examples of **A** Vaccination coverage and equity and **B **Facility-based (institutional) delivery by wealth quintile. *Source: Victora et al, *[45]

We found these outcomes were high overall for most vaccination EBIs. There was high reach across most countries for measles vaccination (86% in Bangladesh in 2014, 90% in Nepal in 2016, 92% in Peru in 2015, 95% in Rwanda in 2015, and 88% in Senegal in 2016). With a priority on reach and coverage, Rwanda leveraged rapid scale-up, aiming for full coverage, and prioritizing the use of global versus local data for decision-making as implementation strategies, with coverage reaching 97% 1 year after introduction. There was subnational variability in some countries based on geography and income (see Fig. 2a). A variety of strategies, and particularly community-based delivery, contributed to the successful implementation overall. Challenges for reach included culture, geography, and national prioritization.

#### Equity

We found substantial progress towards increased equity in all six countries, with equity gaps for many EBIs diminishing nationally. Nevertheless, for some EBIs including four or more visits for antenatal care, vaccinations, FBD, and oral rehydration therapy, there was substantial variation subnationally (see Fig. 2a and b) [32]. Several countries had challenges with geographic inequity for antenatal care, delivery by a skilled provider, and FBD, including Bangladesh, Ethiopia, Nepal, Peru, and Senegal. In Ethiopia and Bangladesh, disparities in outcomes by wealth or geography persisted across EBIs. Challenges to implement included geographic access, culture and beliefs (acceptability), and civil unrest. Strategies to increase equity included data use for prioritization, rapid scale up, building on CHW programs and community-based care delivery, engagement and education of community, and multisectoral collaboration.

To reduce U5M due to malaria, Bangladesh targeted equity and reach as outcomes, using strategies such as rapid scale up, when focusing on 13 malaria-endemic districts in the northeast and southeast (rather than the entire country) for its insecticide-treated nets program. This implementation resulted in achieving high reach, with 92% of U5 children sleeping under an insecticide-treated net in the southeast and 87% in the northeast between 2008 and 2011. Similarly, for prioritization for indoor residual spraying for malaria prevention implementation, Senegal employed strategies including focus on equity and data use in prioritizing areas with the highest need versus full coverage, leading to limited but targeted coverage from 3% in 2007 to 12% in 2016.

#### Sustainability

We found sustainability was high when countries focused early on integrating EBIs into primary care systems – such as integrating vaccination EBIs into routine immunization schedules. Additional strategies, including integration of EBIs into national budgets or work to build community trust in the health system and commitment from national leadership through financial investment, contributed to the outcome of sustainability. Community trust and commitment from national leadership were important in achieving sustainability in Rwanda which integrated the monitoring of coverage and the maintenance of the national supply chain. In doing so, Rwanda secured the counterpart funding in the annual national budget for all vaccines for U5. In contrast, the lack of long-term government financing threatened sustainability. For example, in Peru’s decentralized vaccination program, regions were responsible for the costs of distributing the rotavirus vaccine, but some did not receive the necessary funding allocations to do so.

## Discussion

Our cross-country analysis using existing case studies compared how Nepal, Rwanda, Bangladesh, Senegal, Peru, and Ethiopia chose, implemented, and adapted strategies to mitigate barrier or leverage facilitator contextual factors, and improve implementation outcomes, in order to implement health system-delivered EBIs to reduce amenable U5M. We found that these six countries were often strategic in how they chose and implemented EBIs known to reduce amenable U5M. They often used a common pathway of Exploration, Preparation, Implementation, Adaptation, and Sustainment. They typically made use of locally or globally generated data to identify the needs, select the EBIs, choose implementation strategies, adapt those strategies to their local context, and sustain implementation of the EBIs and adapted strategies.

Contextual factors had a substantial impact in the implementation of EBIs to reduce amenable U5M, and in broader reduction of U5M. Across all six countries, national priority for health (including for U5M), leadership and governance, and a culture of accountability were critical factors which either preexisted or were developed during the study period to establish a local context in which implementation of EBIs could be successful. The role of national priority for health as an important potential facilitator supports Moucheraud et al. who found political commitment and strong leadership aided progress towards MDG 4 – and that conversely a *lack* of political commitment was a key factor to lack of attention for neonatal health during the same period [7]. Similarly, Hailemariam et al. found national leadership and accountability can also increase likelihood of sustainment [46], while a lack of accountability for results could be a barrier to accessing and utilizing learning from others [47].

Our findings support and expand on previous studies that have found community health systems and structure, and in particular existence and use of CHW programs and focus on primary care, to be a critical facilitating contextual factor for the reduction of amenable U5M [48–50]. Increasingly, CHWs are found to play a critical role in supporting community health systems and structure – and yet their less formal position within the health sector and the quality of service they provide are commonly cited as a challenge to their success [48, 51, 52]. Further challenges include the potential for overburdening CHWs by continuously adding to their work as health programs increase, inadequate training, and high turnover [49, 51]. In a recent article on coverage equity by delivery channel, community-based delivery of interventions, largely delivered by CHWs – including education and promotion of vaccination and case management for common diseases – were found to be among the most equitable mode of delivery [50]. Use of CHWs is associated with significant increases in access to care especially in rural areas [51]. Community health systems which used CHWs and garnered community trust have contributed to strengthening interventions to reduce U5M, integrating interventions, and increasing community engagement [49].

The contributions of non-health system and cross-system factors including female empowerment, national infrastructure and systems strengthening, and economic growth were important in all of the six countries studied. These findings are consistent with other studies which have found significant contributions from non-health system interventions including female empowerment and stunting reduction [2, 4] and, critically during the MDG period, economic growth [7]. Similarly, a project for electrification of health centers in Ghana and Uganda was linked to improved service availability and use – as well as supporting EBIs including through appropriate storage of vaccines [53].

We found that major change can be brought where there are major barriers, when countries choose and implement strategies which either address these directly or provide opportunities for circumventing them. For example, Nepal made significant advances in implementing EBIs despite ongoing conflict, and a challenging mountainous geography that limited access to healthcare for rural and remote populations. Rwanda, despite having a gross domestic product of just $219 per capita in 2000 [54], was able to leverage donor funding to successfully implement EBIs. Ethiopia overcame challenges due to a large population size by rolling out a large-scale community Health Extension Program to increase coverage. Other cross-country studies have similarly noted that reforms have often been catalyzed by conflict, crisis, or new global opportunities (such as GAVI) – which can provide both the need and motivation to enact change, as well as the opportunity to challenge the existing context [47]. The six countries were often able to overcome major obstacles to implement large-scale transformation, a reminder to all of what can be achieved in contexts with substantial challenging contextual factors.

We found a number of implementation strategies which were common across countries and most EBIs. Knowing when to utilize and when to adapt strategies can be as important as the actual choice of strategy [19], and further, adaptation can also increase likelihood of sustainment [46]. Community-based care in these six countries was often delivered by CHWs at no cost to families, meaning that this was also often a strategy that could increase equity [50]. Engagement of community, and long-term partnerships, are important to the adaptation, implementation, and sustainability of EBIs [55]. Multisectoral collaboration and stakeholder and donor engagement and coordination were critical across countries and phases of EPIAS, and closely tied to other strategies including use of data. Developing stakeholder interrelationships was similarly identified in a study of key strategies and outcomes for newborn care EBIs [56]. Our findings support literature that shows strategies that actively engage stakeholders may be more effective than more passive strategies such as adoption of global guidelines [55, 57, 58].

Adaptation of strategies can be an essential way to increase implementation outcomes including acceptability, feasibility, equity, and sustainability. According to Lewis and colleagues, determinants – or contextual factors – can help with understanding why a strategy did or did not have its intended outcome. Beyond that though, it is understanding the *mechanism* which will help explain how a strategy had an effect, or not [59]. For example, acceptability of an EBI such as FBD may be very low in culturally sensitive environments across different countries [60]. A country’s strategy of adaptation to allow traditional birthing positions likely operated through the mechanism of compromise or accommodation to increase the willingness of pregnant women to deliver in facilities, ultimately leading to the outcome of increased acceptability. As reflected in Peru’s case of cultural adaptation of FBD [61], the design and implementation of the interventions may need to be adapted to local cultural practices and beliefs to ensure community acceptability, uptake, and increase in coverage of the services [60].

Implementation research studies more typically focus on outcomes including adoption, fidelity, sustainability, and cost [55], but depending on the setting data for some of these measures can be challenging to come by. We found that feasibility of EBIs, for example, was influenced by a number of factors including the nature of the intervention, community acceptability, and leadership involvement. Community IMCI, where CHWs treat common childhood illness at community level, is one such example. As found in other studies, CHWs tend to be accepted by their neighbors as they are commonly selected by communities they live among [49, 62], which further increases the likelihood of outcomes including acceptability, feasibility, and equity. Implementation of this program mainly requires leadership follow-up and use of small budget, which helps with feasibility of its rollout [63, 64]. In addition, sustainability of EBIs such as pneumococcal conjugate vaccine and rotavirus vaccination was mainly dependent on funding availability from both partners and governments. In case partners either reduced or stopped their funding, governments had to integrate the vaccine delivery into the existing systems and national budget, possibly due to GAVI and WHO requirements that countries receiving support for their immunization programs have financial sustainability plans for immunization services [65, 66].

In each of these countries, remaining challenges include expanding and sustaining coverage in areas where the EBIs were still not fully and effectively implemented, and challenges in quality of care, both experiential and technical. The six countries focused more on access and coverage and collection of data than on effective coverage and ensuring and sustaining performance of an EBI’s implementation to reduce the causes of death for U5M. More recent UN data show that all six countries have continued to decrease U5M, and between 2015 and 2020 have done so with a greater percentage change than global averages [67, 68]. While these trends suggest ongoing progress, research focusing on country progress towards achieving Sustainable Development Goal U5M targets has reflected on the unequal progress on U5M subnationally in countries, suggesting that more work will be necessary to maintain this progress for all populations [69–72].

Countries confront ongoing challenges in planning, monitoring, and evaluation of EBIs. The future sustainability of implementing EBIs to reduce U5M faces health sector threats such as the COVID-19 pandemic or potential decreases in the availability of funds and global support. However, learnings from implementation strategies adopted for U5M EBIs, and increased understanding of critical contextual factors, have the potential to help increase resilience in the face of these threats moving forward. This work will benefit from future research to better understand whether and how countries have maintained their successful approaches during the COVID-19 pandemic.

The identification of common implementation strategies and contextual factors that can facilitate or impede the strategies use for an EBI’s successful implementation; the exploration of a common pathway to implementation; and the overall implementation research tools to understand the “how” and the “why” behind countries’ success, continue to be extremely relevant as we arrive at the midpoint of the Sustainable Development Goals. Many countries are continuing to work to achieve gains in reducing under-5 and neonatal mortality, and there is value to decision-makers in countries trying to replicate the successes and efforts in other countries in having access to transferable lessons learned, information of common implementation strategies, and other resources developed by countries that have experienced success.

Our study had a number of limitations. The most significant limitation was related to the scope of funded work to only look at successful countries, without the important comparison of factors in countries that did not achieve these major reductions in U5M. While the cross-case analysis methodology strengthens the evidence for the successful strategies identified, without the comparison to strategies and factors in countries with less success in reducing U5M, these conclusions need to be interpreted with caution, and work to replicate these case study methods in these other countries with more limited reductions in U5M is needed. Other limitations included limited availability of data or other information from multiple sources on outcomes, context, and strategies. There were limitations in the numbers and range of our key informant interviews, which were retrospective. This meant we could not do contribution analysis or claim attribution or causality, which would have also required more extensive quantitative data. We were limited in our ability to conduct research into some factors associated with U5M such as malnutrition, and the scope of our work was limited to amenable U5M and health system-delivered EBIs – which meant that the not-insubstantial contribution of non-health-sector interventions was beyond the scope of this study.

## Conclusion

The six countries included in this analysis achieved significant reductions in amenable U5M. They used similar approaches to implementation of health system EBIs. They made national and local adaptations while going through similar journeys in selecting appropriate EBIs, identifying and leveraging or addressing contextual factors, and adopting and adapting implementation strategies. Cross-cutting strategies implemented across all or most of these countries included data use for decision-making, multisectoral collaboration, and building and leveraging existing primary healthcare systems to integrate new EBI delivery into existing systems including CHWs. These strategies are all adoptable by other countries and contexts and can be adapted to address or leverage identified contextual factors before or during implementation. Countries can use this work to identify, analyze, and build on their contextual factors in recognition of where adaptations are needed to increase effective coverage and ensure a more resilient response in continuing to reduce amenable U5M and contribute to strengthening public health system delivery now and in the future.

### Supplementary Information

**Additional file 1. **a. List of infant and child evidence-based interventions, and b. List of neonatal evidence-based interventions.

**Additional file 2. **Example Exemplars in Under 5 Mortality key informant interview guide.

**Additional file 3. **Interaction of selected contextual factors, implementation strategies, and targeted outcomes, and associated Exploration, Preparation, Implementation, Adaptation, and Sustainment (EPIAS) stage.

## Data Availability

The case studies analyzed for this cross-country analysis are available from https://www.exemplars.health/topics/under-five-mortality. Bangladesh: https://www.exemplars.health/-/media/files/egh/resources/underfive-mortality/bangladesh/bangladesh-case-study-_-final-28082020.pdf Ethiopia: https://www.exemplars.health/-/media/files/egh/resources/underfive-mortality/ethiopia/ethiopia-case-study-_-final-_10042020.pdf Nepal: https://www.exemplars.health/-/media/files/egh/resources/underfive-mortality/nepal/nepal-case-study_-final-_10042020.pdf Peru: https://www.exemplars.health/-/media/files/egh/resources/underfive-mortality/peru/peru-case-study-_-final-28082020.pdf Rwanda: https://www.exemplars.health/-/media/files/egh/resources/underfive-mortality/rwanda/rwanda-case-study_-final-28082020.pdf Senegal: https://www.exemplars.health/-/media/files/egh/resources/underfive-mortality/senegal/senegal-case-study-_-final-28082020.pdf All quantitative data were from publicly available sources. Qualitative data access is restricted to users with appropriate ethics approval from the committees listed in the Ethical Considerations section. A reader or reviewer may apply to the authors for access by providing a written description of background, reasons, and intended use. If the methodology does not violate the condition of informed consent under which the interview was conducted, and the proposal approved by UGHE and other relevant ethics boards, the user can obtain the data from the corresponding author, and include one of the authors in the project and analysis.

## Abbreviations

CHW: Community health worker
EBI: Evidence-based intervention
EPIAS: Exploration, Preparation, Implementation, Adaptation, Sustainment
FBD: Facility-based delivery
IMCI: Integrated management of childhood illness
KI: Key informant
MDG: Millennium Development Goal
MOH: Ministry of Health
PMTCT: Prevention of mother-to-child transmission of HIV
U5M: Under-5 mortality
