# Supplementary material for: Cross-country analysis of contextual factors and implementation strategies in under-5 mortality reduction in six low- and middle-income countries 2000–2015
Source: BMC Pediatr. 2024 Feb 28;23(Suppl 1):652. doi: 10.1186/s12887-023-03906-5 (PMC10900537; doi:10.1186/s12887-023-03906-5)
Supplement: Supplementary file 1 — Additional file 1. a. List of infant and child evidence-based interventions, and b. List of neonatal evidence-based interventions. [file 12887_2023_3906_MOESM1_ESM.pdf]

Additional File 1a. List of infant and child evidence-based interventions

Cause of Death	EBI	
**Lower respiratory infections**	Antibiotic treatment	
	Vaccination: PCV	
	Vaccination: Hib	
	Community-based management	
	Facility-based management	
**Diarrheal diseases**	Oral rehydration therapy	
	Zinc supplementation	
	Vaccination: Rotavirus	
	Community-based management	
	Facility-based management	
**Malaria**	Antimalarial combination therapy	
	Rapid diagnostic testing	
	Insecticide-treated nets	
	Indoor residual spray	
	Intermittent preventative therapy for high-risk groups	
	Community-based management	
	Facility-based management	
**Measles**	Vaccination: Measles	
	Vitamin A supplementation (prior to vaccination)	
**Malnutrition**	Exclusive breastfeeding for six months	
	Continued breastfeeding and complementary feeding after six months	
	Vitamin A supplementation	
	Management of severe acute malnutrition (ready-to-use food, rehydration, antibiotics)	
**HIV**	ARV treatment for infants and children	
	HIV testing of children born to HIV+ mothers	
	Prevention of mother-to-child transmission	Early diagnosis of pregnant women (or pre-pregnancy)
		PMTCT treatment for mothers* and post-partum to exposed infants
		Elective C-section for untreated HIV+ mothers**; replacement feeding**
		ARV treatment for mother for life as prevention (started in 2012)
		Exclusive breast feeding
**Meningitis**	Vaccination: PCV meningococcal	
	Vaccination: Hib	
	Vaccination: Meningococcal	
	Antibiotic treatment	
	Chemoprophylaxis during acute outbreaks	
**Other vaccine preventable diseases**	Vaccination: Tetanus	
	Vaccination: Diphtheria	
	Vaccination: Pertussis	
	Vaccination: Polio	

ARV: anti-retroviral therapy; PCV: pneumococcal conjugate vaccine; PMTCT: prevention of mother-to-child transmission of HIV

* No longer recommended (PMTCT versus ART for life)

**No longer recommended for women on ART with suppressed viral load

Additional File 1b. List of neonatal evidence-based interventions

Period of risk	EBI	
**Preconception**	Folic acid supplementation	
**Antenatal**	Tetanus vaccination	
	Malaria prevention and treatment	Intermittent presumptive treatment
		Insecticide-treated bed nets
	Iodine supplementation (in endemic iodine deficient settings)	
	4 or more antenatal visits (ANC4)	
	Prevention and treatment of preeclampsia and eclampsia	Antihypertensive treatment for severe hypertension
		Magnesium sulfate
		Early delivery
**Intrapartum**	Antibiotics for preterm premature rupture of membranes	
	Corticosteroids for preterm labor	
	C-section for breech or obstructed labor	
	Active management of delivery (including partograph)	
	Clean delivery practices (incl. clean cord-cutting)	
	Trained birth attendant	
	Facility-based delivery	
	Basic emergency obstetric and newborn care (BEmONC)	
	Comprehensive emergency obstetric and newborn care (CEmONC)	
	Timely transport for higher level care for mother	
**Postnatal**	Newborn resuscitation	
	Immediate breastfeeding	
	Prevention and management of hypothermia	Immediate drying and wrapping
		Delayed bathing
		Skin-to-skin
		Baby warming
	Kangaroo care for LBW/prematurity	
	Timely transport for higher level care for mother	
	Post-partum visits to identify danger signs and provide active referral	
	Antibiotics for suspected or confirmed infection	
	Surfactant therapy for respiratory distress syndrome and prematurity	
	Neonatal intensive care units (equipped, trained staff, standards and protocols established and followed)	
