# Supplementary material for: Cross-country analysis of contextual factors and implementation strategies in under-5 mortality reduction in six low- and middle-income countries 2000–2015
Source: BMC Pediatr. 2024 Feb 28;23(Suppl 1):652. doi: 10.1186/s12887-023-03906-5 (PMC10900537; doi:10.1186/s12887-023-03906-5)
Supplement: Supplementary file 2 — Additional file 2. Example Exemplars in Under 5 Mortality key informant interview guide. [file 12887_2023_3906_MOESM2_ESM.pdf]

Additional File 2. Example Exemplars in Under 5 Mortality Key Informant Interview Guide

Interviewee Information
Name:
Date:
Interviewer(s):
____ Phone ____ In person

Informed consent received: ____ Yes ____ No (if no-end interview)
Recording consent received: ____ Yes ____ No

1. What position(s) did you hold starting in 2000? And after?

year	Position/typology*	relevance

***National level, MOH-across divisions, MOH-specific divisions, Implementers (EBI or area of focus if relevant) External donors, Others

If pertinent, prior to 2000

2. What is your current position?

3. Please describe the role you and your organization played in the work to reduce U5M in Senegal
   a. Probe: location, area of focus, duration, age range, level of health system, specific EBI or COD

Senegal has made remarkable progress in dropping the rate of U5 mortality since the year 2000. We will be focusing on the time period between ____________________ (if they were only involved for part of the 15 years)

4. Thinking broadly, what do you think were key facilitators in Senegal that helped to enable this success both in 2000 and over time?

   Probe: Ministry leadership
   Organizational structure
   Interaction with other ministries
   Culture and environment in the ministry
   Donors
   Engagement of stakeholders
   National level factors

5. What were some barriers that challenged the ability to start or continue the work to reduce U5M?

   Probe: Ministry leadership
   Organizational structure
   Needs from other ministries
   Culture and environment in the ministry
   Donors/funding
   Engagement of stakeholders
   National level factors

   Probe: In 2005? 2010? Over the last 5 years?

6. What changes were made to overcome these?

7. What other factors contributed to the reduction of U5M, initially in 2000 and over time?

   Probe: Partnerships
   Culture
   Learning
   Accountability
   Stakeholders
   Leadership

8. Where are areas where there are new or remaining challenges in U5M?

COD or EBI we are interviewing for: ______________________________

9. How was U5M recognized as a priority problem for work at the MOH?

Probe: Data and other sources of information
Funding opportunities
Other input from within country, donors
Comparing with other countries
Community/stakeholder engagement
National government

Probe: When was a focus on Neonatal mortality introduced (if it was)? Why?

10. What other specific influences led to national and ministry decisions to address U5M and specific priorities such as age groups or cause of death or areas of the country (versus or in addition to other problems)?

- Probe:
  - Evidence
    - (overall and specific causes of death)
    - New interventions
  - Leadership at national level
  - Funding/donors
  - Legislation/policies
  - Mission/values of the MOH, of the country

11. What facilitated your ability to prioritize and get resources to address U5M overall and targeted areas (COD or age group)?

a. Probe:
   i. donor funding
   ii. national leadership
   iii. partnership with Ministry of finance

12. **(Show the list of EBIs) In your opinion, which were the top 3 EBIs that contributed to the success of reducing U5M overall since 2000?**

***EBI1:* ________________________**

a. How did you choose this one?
   Probe: Evidence of need, evidence of effectiveness
   Funding,
   national government priority
   stakeholders priority (community, partners, donors)

b. How did you prepare to implement this intervention?
   Probe:
   Adaptation of intervention and implementation, use of pilots
   Stakeholder engagement (partners, community, other elements of government)

c. What helped you implement it?
   i. how it was started (ilot, or not, etc)
   ii. evidence available on effectiveness
   iii. leadership buy-in
   iv. stakeholder support/engagement process
   v. accountability,

d. What were the challenges?
   i. Funding
   ii. Structure and strength of the health system
   iii. Legislation
   iv. Community
   v. quality
   vi. other

e. What did you need to change once you had started?
   Probe: Intervention components vs implementation, why it was changed, any

f. Overall how effective was the implementation of the intervention
   i. Probe:
      1. Reach-overall, equity
      2. Acceptability by community and providers
      3. Fidelity (quality)
      4. Feasibility ex. Cost, ability to implement
      5. Effectiveness

g. Have you been able to reach scale and sustain the intervention and change?
   i. What are the challenges (if any) to sustaining this success

***EBI2:* ________________________**

a. How did you choose this one?
   Probe: Evidence of need, evidence of effectiveness
   Funding,
   national government priority
   stakeholders priority (community, partners, donors)

b. How did you prepare to implement this intervention?
   Probe:
   Adaptation of intervention and implementation, use of pilots
   Stakeholder engagement (partners, community, other elements of government)

c. What helped you implement it?
   ii. how it was started (ilot, or not, etc)
   iii. evidence available on effectiveness
   iv. leadership buy-in
   v. stakeholder support/engagement process
   vi. accountability,

d. What were the challenges?
   vii. Funding
   viii. Structure and strength of the health system
   ix. Legislation
   x. Community
   xi. quality
   xii. other

e. What did you need to change once you had started?

Probe: Intervention components vs implementation, why it was changed, any

f. Overall how effective was the implementation of the intervention
   xiii. Probe:
      1. Reach-overall, equity
      2. Acceptability by community and providers
      3. Fidelity (quality)
      4. Feasibility ex. Cost, ability to implement
      5. Effectiveness

g. Have you been able to reach scale and sustain the intervention and change?
   What are the challenges (if any) to sustaining this success

***EBI 3:* ______________________________**

a. How did you choose this one?
   Probe
   - Evidence of need, evidence of effectiveness
   - Funding,
   - national government priority
   - stakeholders priority (community, partners, donors)

b. How did you prepare to implement this intervention?
   Probe:
      Adaptation of intervention and implementation, use of pilots
      Stakeholder engagement (partners, community, other elements of government)

c. What helped you implement it?
   - how it was started (ilot, or not, etc)
   - evidence available on effectiveness
   - leadership buy-in
   - stakeholder support/engagement process
   - accountability,

d. What were the challenges?
   - Funding
   - Structure and strength of the health system
   - Legislation
   - Community
   - quality
   - other

e. What did you need to change once you had started?
   Probe: Intervention components vs implementation, why it was changed, any

f. Overall how effective was the implementation of the intervention
   Probe:
- Reach-overall, equity
- Acceptability by community and providers
- Fidelity (quality)
- Feasibility ex. Cost, ability to implement
- Effectiveness

g. Have you been able to reach scale and sustain the intervention and change?
   What are the challenges (if any) to sustaining this success

13. If U5M was already dropping by 2000, ask for general EBIs and factors which were associated with the decline prior to 2000

14. In your opinion, what were 1-2 EBIs that were not effective or less effective than expected in contributing to reducing U5M overall or neonatal mortality specifically?

***EBI1:* __________________________**

a. How did you choose this one?
   Probe: Evidence of need, evidence of effectiveness
      Funding,
      national government priority
      stakeholders priority (community, partners, donors)

b. What do you think made it ineffective?
   Probe: Preparation – adaptation, partners, engagement
   Probe: Implementation – strategy, start and scale, leadership, accountability, sustainment, culture, ineffectiveness
   Probe: Outcomes – Reach, acceptability, adoption, feasibility, cost, fidelity

c. What would you change if you did it again?
   Probe: Intervention components and/or implementation strategy

***EBI2:* __________________________**

a. How did you choose this one?
   Probe: Evidence of need, evidence of effectiveness
      Funding,
      national government priority
      stakeholders priority (community, partners, donors)

b. What do you think made it ineffective?
   Probe: Preparation – adaptation, partners, engagement
   Probe: Implementation – strategy, start and scale, leadership, accountability, sustainment, culture, ineffectiveness
   Probe: Outcomes – Reach, acceptability, adoption, feasibility, cost, fidelity

c. What would you change if you did it again?
   Probe: Intervention components and/or implementation strategy

15. Thinking broadly, what do you think were key facilitators at the Ministry of Health that helped to enable this success since 2000 (note the timeframe)?
   Probe: Ministry leadership
      Organizational structure
      Interaction with other ministries
      Culture and environment in the ministry
      Donors
      Engagement of stakeholders
      National level factors

16. What were some barriers that challenged your ability in the work to reduce U5M (note the time frame)?

Probe: Ministry leadership
Organizational structure
Needs from other ministries
Culture and environment in the ministry
Donors/funding
Engagement of stakeholders
National level factors

Probe: Who held your position before you?
Who held your position after you?

17. What changes were made to overcome these?

18. Overall, how did you know if work to reduce U5M and/or NMR was on-track or off-track?

19. What other factors outside of the MOH contributed to the reduction of U5M since 2000? How has that differed for NMR?

Probe:

- Partnerships (probe-with whom-implementers, private sector, donors others)
- Other interventions (ex. Nutrition, WASH, economic development, education)
- Learning at the MOH
- Accountability in the health sector
- Governance and Leadership (national or local)

20. Where are areas where there are new or remaining challenges in U5M?
Probe: age (NMR), change in COD, equity/coverage, other?

21. From the lessons learned in Senegal, what would be knowledge and lessons which you think could be transferable (with adaptation) to other countries trying to reduce U5M generally including neonatal mortality and how would you suggest they implement them?

Contextual factors:
Governance
Accountability
Donor coordination
Financial
Prioritization/culture (ex. Equity)
HMIS/data use

EBIs and how they were chosen and implemented
Policy which need to be in place
Stakeholder engagement
How decisions were made
Interaction/coordination with other ministries
Role of private sector
Role of research

22. If there is an area not well covered in the desk review-ask if there any really critical reports or sources of information that would help us better understand the progress and challenges of reducing U5M since 2000 for ____________________?

23. Who else should we speak to better understand the reductions in U5M in Senegal?

Non-neonatal EBIs Interventions delivered through the Health System		
**Cause of Death**	**Evidence-Based Interventions**	
Lower respiratory infections	Antibiotic treatment	
	Vaccination: PCV	
	Vaccination: Hib	
	Community-based management	
	Facility-based management	
Diarrheal diseases	Oral rehydration therapy	
	Zinc supplementation	
	Vaccination: Rotavirus	
	Community-based management	
	Facility-based management	
	WASH (prevention)*	
Malaria	Antimalarial combination therapy	
	Rapid diagnostic testing	
	Insecticide-treated nets	
	Indoor residual spray	
	Intermittent preventative therapy for high-risk groups	
	Community-based management	
	Facility-based management	
Measles	Vaccination: Measles	
	Vitamin A supplementation (prior to vaccination)	
Malnutrition	Promotion and support of exclusive breastfeeding for 6 months	
	Promotion of continued breastfeeding and complementary feeding after 6 mos	
	Vitamin A supplementation	
	Management of severe acute malnutrition (ready-to-use food, rehydration, antibiotics)	
	Broader nutrition efforts (prevention)*	
HIV	Antiretroviral treatment for infants and children	
	HIV testing of children born to HIV+ mothers	
	Prevention of mother-to-child transmission	Early diagnosis of pregnant women (or pre-pregnancy)
		PMTCT treatment for mothers** and post-partum to exposed infants
		Elective C-section for HIV+ mothers***; replacement feeding***
		ART for mother for life (B+) (started in 2012)
		Exclusive breast feeding
Meningitis	Vaccination: PCV	
	Vaccination: Hib	
	Vaccination: Meningococcal	
	Antibiotic treatment	
	Chemoprophylaxis during acute outbreaks	
Other vaccine preventable diseases	Vaccination: Tetanus	
	Vaccination: Diphtheria	
	Vaccination: Pertussis	
	Vaccination: Polio	
General	Vitamin A	
Syphilis	Screening of pregnant women	
	Treatment with penicillin (pregnant women, exposed infants)	

*: broader initiatives; will note coverage ** no longer recommended ***No longer recommended for women on ART with suppressed VL

Neonatal-Specific Evidence-Based Interventions delivered through the Health System		
**Period of Risk**	**Evidence-Based Intervention**	
Preconception	Folic acid supplementation	
Antenatal	Tetanus vaccination	
	Malaria prevention and treatment	Intermittent presumptive treatment
		ITNs
	Iodine supplementation (in endemic iodine deficient settings)	
	4 or more antenatal visits (ANC4)	
	Prevention and treatment of preeclampsia and eclampsia	Antihypertensive treatment for severe hypertension
		Magnesium sulfate
		Early delivery
Intrapartum	Antibiotics for PPROM	
	Corticosteroids for preterm labor	
	C-section for breech or obstructed labor	
	Active management of delivery (including partograph)	
	Clean delivery practices (incl. clean cord-cutting)	
	Trained birth attendant	
	Facility-based delivery	
	Access to Basic emergency obstetric and newborn care (BEmONC)	
	Access to Comprehensive emergency obstetric and newborn care (CEmONC)	
	Timely transport for higher level care for mother (can be postnatal period as well)	
Postnatal	Newborn resuscitation	
	Immediate breastfeeding	
	Prevention and management of hypothermia	Immediate drying and wrapping
		Delayed bathing
		Immediate Skin-to-skin
		Baby warming
	Kangaroo care for LBW/premature	
	Timely transport for higher level care for mother	
	Timely transport for higher level care for neonate	
	Post-partum visits to identify danger signs and provide active referral	
	Antibiotics for suspected or confirmed infection	
	NICUs (equipped, trained staff, standards/protocols established and followed)	

Not found but could be considered: Prevention of premature labor

Selected Contextual Factors Associated with Reduced Risk of U5M:
Maternal education
Birth spacing and reduced fertility rates
General nutrition programs (maternal and child)
Expansion of human resources
Increased access (geographic, financial)
Improved quality of care
