# Supplementary material for: Cross-country analysis of contextual factors and implementation strategies in under-5 mortality reduction in six low- and middle-income countries 2000–2015
Source: BMC Pediatr. 2024 Feb 28;23(Suppl 1):652. doi: 10.1186/s12887-023-03906-5 (PMC10900537; doi:10.1186/s12887-023-03906-5)
Supplement: Supplementary file 3 — Additional file 3. Interaction of selected contextual factors, implementation strategies, and targeted outcomes, and associated Exploration, Preparation, Implementation, Adaptation, and Sustainment (EPIAS) stage. [file 12887_2023_3906_MOESM3_ESM.pdf]

Additional File 3. Interaction of selected contextual factors, implementation strategies, and targeted outcomes, and associated Exploration, Preparation, Implementation, Adaptation, and Sustainment (EPIAS) stage

**Contextual factors, level, and where they were a facilitator or barrier**	**Contextual factor role as facilitator or barrier**	**EPIAS stage where contextual factors were important**	**Implementation strategies chosen to address or leverage contextual factor (and associated EPIAS stage)**	**Outcomes targeted**[1]
Donor funding priorities and availability<br>(Global)<br>*Facilitator in all countries, though could be barrier at times*	Facilitator: If present and coordinated, resulted in more efficient and sustained implementation.<br>Barrier: When time limited or not aligned, could threaten a sustainable implementation or one which is not aligned with national plans.	Exploration<br>Implementation<br>Sustainment	Leveraging donor support *(EPIS)*<br>Donor coordination *(EPIA)*<br>Government financing *(PIAS)*	Feasibility
Geography<br>(National/subnational)<br>*Barrier in most but adapted to,*<br>*Facilitator in Rwanda,*<br>*No effect in Peru*	Facilitator: If a facilitator, leads to better access with implications for equity, reach, and coverage.<br>Barrier: If a barrier, limits equity, reach and coverage, and a focus on equity (ex. subsidized transport), community-based care delivery, phased or limited scale-up targeting the most in need are required to address.	Preparation<br>Implementation<br>Adaptation	Focus on equity *(EPIAS)*<br>Rapid vs. phased vs. limited scale-up *(IA)*<br>Community-based care delivery *(PIAS)*<br>Leveraging in-country partner support *(EPIAS)*	Acceptability<br>Feasibility<br>Effectiveness<br>Cost<br>Coverage<br>Reach<br>Fidelity<br>Equity<br>Sustainability
Culture of donor and partner coordination<br>(MOH/health systems)<br>*Facilitator in most,*<br>*Both facilitator and barrier in Senegal*	Facilitator: If present, allows for coordinated, frequent engagement and leveraging of donor and partner resources required for efficient implementation.<br>Barrier: Otherwise limits engagement and leveraging resources as well as alignment of donors and partners which impact sustainability of implementation.	Preparation<br>Implementation	Leveraging international and in-country partner and donor support *(EPIAS)*<br>Donor and implementing partner coordination (EBI-specific) *(EPIAS)*<br>Stakeholder engagement (international) *(EPIAS)*	Appropriateness<br>Acceptability<br>Feasibility<br>Reach<br>Coverage<br>Fidelity<br>Sustainability<br>Equity

[1] *Targeted* means that it influenced the choice of the strategy, versus the strategy improving that outcome if done well.

**Contextual factors, level, and where they were a facilitator or barrier**	**Contextual factor role as facilitator or barrier**	**EPIAS stage where contextual factors were important**	**Implementation strategies chosen to address or leverage contextual factor (and associated EPIAS stage)**	**Outcomes targeted**[1]
Culture and capacity of data use (MOH/health systems)<br>*Facilitator in all*	<u>Facilitator</u>: If present, supports data use for decision-making at all stages of EPIAS required for efficient implementation.<br><u>Barrier</u>: If a barrier (ex. limited capacity), limits data use for decision-making required for timely and efficient implementation.	Exploration<br>Preparation<br>Implementation<br>Adaptation<br>Sustainment	Data use for decision-making *(EPIAS)*<br>Rapid and early adoption of innovations *(PI)*<br>Small-scale testing *(PIA)*<br>Data use to understand disease burden *(EPI)*<br>Data use for prioritization *(EPIAS)*<br>Supportive supervision and mentoring *(IAS)*<br>Use of surveillance including monitoring and evaluation *(PIAS)*<br>Data use for adaptation *(IAS)*<br>Data systems strengthening *(EPIS)*<br>Focus on equity *(EPIAS)*<br>Focus on improving quality *(IAS)*<br>Prioritization of neonatal mortality *(PIA)*	Acceptability<br>Effectiveness<br>Coverage<br>Reach<br>Fidelity<br>Sustainability<br>Cost<br>Equity
Culture and beliefs (Community/family/ individual)<br>*Facilitator in Rwanda, Senegal*<br>*Barrier in Nepal, Ethiopia, Peru*<br>*Both in Bangladesh*	<u>Facilitator</u>: If favorable/supportive culture and beliefs are present, improves access and acceptability.<br><u>Barrier</u>: Otherwise limits acceptability, feasibility, reach, and many other outcomes unless strategies including stakeholder engagement and community sensitization are adopted.	Preparation<br>Implementation<br>Adaptation	Stakeholder engagement (local) *(EPIAS)*<br>Community education and sensitization *(PIA)*<br>Community engagement *(PIA)*<br>Data use for prioritization *(EPIAS)*<br>Focus on equity *(EPIAS)*	Acceptability<br>Feasibility<br>Reach<br>Fidelity<br>Sustainability<br>Equity
